# Development of microsatellite markers for the invasive mosquito *Aedes koreicus*

**DOI:** 10.1186/s13071-023-05823-z

**Published:** 2023-07-06

**Authors:** Laura Soresinetti, Irene Arnoldi, Agata Negri, Giovanni Naro, Alice Michelutti, Fabrizio Montarsi, Andrea Mosca, Claudio Bandi, Paolo Gabrieli, Sara Epis

**Affiliations:** 1 Department of Biosciences and Pediatric Clinical Research Center “Romeo Ed Enrica Invernizzi”, University of Milan, 20133 Milan, Italy; 2 Department of Biology and Biotechnology, University of Pavia, 27100 Pavia, Italy; 3 University School of Advanced Studies Pavia, IUSS, 27100 Pavia, Italy; 4 Italian Malaria Network, Inter University Center for Malaria Research, University of Milan, 20133 Milan, Italy; 5 Department of Environmental Biology, Sapienza University of Rome, Via Dei Sardi 70, 00185 Rome, Italy; 6 Istituto Zooprofilattico Sperimentale Delle Venezie, 35020 Legnaro, Italy; 7 Istituto Per Le Piante da Legno E L ’Ambiente, I.P.L.A. S.P.A, 10132 Turin, Italy

**Keywords:** Invasive mosquitoes, *Aedes* mosquitoes, Simple sequence repeats (SSRs), Population genetics, Monitoring

## Abstract

**Background:**

*Aedes koreicus* is a mosquito species native to East Asia which has recently invaded several countries in Europe. In Italy, this mosquito was first detected in the North-East in 2011 and is now widely distributed in the entire northern part of the country. The development of specific genetic markers, such as microsatellites, is necessary to uncover the dispersal routes of this mosquito from its native areas and, eventually, to plan future control interventions.

**Methods:**

Available raw sequences of genomic DNA of *Ae. koreicus* were screened in silico using BLASTn to identify possible microsatellite-containing sequences. Specific primer pairs were then designed, and their efficiency was determined through polymerase chain reaction (PCR) on 32 individuals of *Ae. koreicus* collected in Italy. PCR conditions were optimised in three multiplex reactions. Genotyping of individual mosquitoes was performed on both single and multiplex PCR reactions. Finally, analysis of intra-population variation was performed to assess the level of polymorphism of the markers.

**Results:**

Mosquito genotyping provided consistent results in both single and multiplex reactions. Out of the 31 microsatellite markers identified in the *Ae. koreicus* genome raw sequences, 11 were polymorphic in the examined mosquito samples.

**Conclusions:**

The results show that the 11 microsatellite markers developed here hold potential for investigating the genetic structure of *Ae. koreicus* populations. These markers could thus represent a novel and useful tool to infer the routes of invasion of this mosquito species into Europe and other non-native areas.

**Graphical Abstract:**

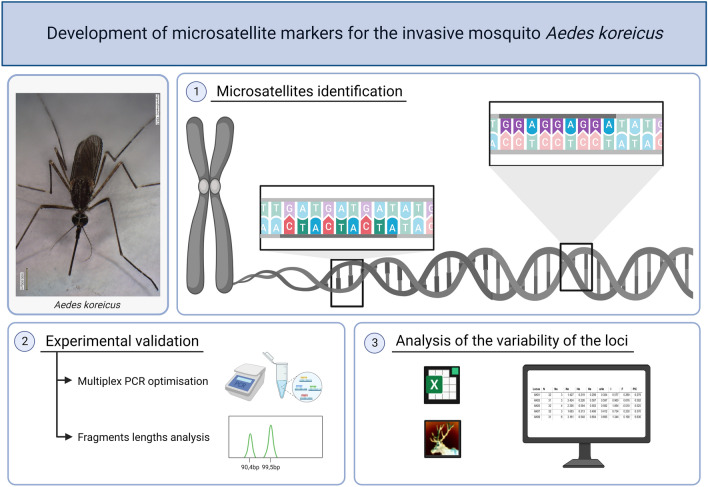

**Supplementary Information:**

The online version contains supplementary material available at 10.1186/s13071-023-05823-z.

## Background

Invasive *Aedes* mosquitoes represent a global concern for public health due to their role as vectors of several pathogens and their ability to colonise new territories [1, 2]. Their dispersal in non-native regions is mainly associated with climate change, and the increasing movement of people and goods (e.g. used tires, ornamental plants, used machineries) [3, 4] that facilitates the importation of alien species into new territories. In the globalisation era, the trade of goods develops into intricate patterns, and the tracking back of invasion routes becomes quite difficult.

In this context, population genetics is a powerful tool: determining the “genetic makeup” of a species can help, for example, in locating the putative origin(s) of multiple introductions, revealing species expansion phenomena, and providing clues on the potential vectorial capacity of different populations of invasive species [5–10].

*Aedes koreicus*, the Korean bush mosquito, is a species native to temperate areas of Asia, particularly Korea, China, and Far East Russia [11]. It was described for the first time outside its endemic territory in Europe, in 2008 in Belgium [12]. Since then, this alien mosquito species has been found in several territories in Southern, Central, and Eastern European countries [13, 14], with the last finding in the Czech Republic in 2022 [15]. The invasiveness success of this Korean mosquito is evident in Italy, where the mosquito populations did not remain confined to relatively small areas, as has instead been observed in other European countries [16]. Indeed, after the first description in Belluno district in 2011 [17], *Ae. koreicus* mosquitoes were collected in all the Italian North-East regions (as recently reviewed in [18, 19]), in the municipality of Genoa (2015) [20], in five districts of the Lombardy region, namely Como (2013), Sondrio (2015), Bergamo (2020), Brescia and Lecco (both in 2021) [14, 21, 22], and in three districts of the Piedmont region, Alessandria, Asti, and Torino (all in 2021) [14, 23].

Despite the large number of reports of *Ae. koreicus* in Europe, and in particular in Italy, possible entry routes into the continent have not been reconstructed so far, nor it is clear how the mosquito spread after its first introduction(s). Filling this knowledge gap is important, considering that the Korean bush mosquito has the potential to further spread in currently uncolonised European countries [14]. Indeed, the study of the genetic structure of populations of *Ae. koreicus* and the understanding of the genetic relatedness between individuals from different locations would permit us to hypothesise how this mosquito spread in Northern Italy, and even in other European countries [24].

To achieve this goal, genetic markers are needed, and microsatellites were selected as the markers of choice. Indeed, simple sequence repeats (SSRs) are characterised by high variability level and relative ease of use at low cost [25]; moreover, microsatellites have been successfully used for revealing the genetic structure of other *Aedes* native and invasive mosquito species, such as *Ae. albopictus*, *Ae. japonicus*, *Ae. taeniorhynchus*, *Ae. polynesiensis*, *Ae. sticticus*, *Ae. fluviatilis*, and *Ae. aegypti* [8, 10, 26–30]. Particularly, SSRs developed for *Ae. japonicus* were indicated as polymorphic also in *Ae. koreicus* [31]. For this reason, we preliminarily assayed these microsatellites using DNA samples from *Ae. koreicus*, but the amplification resulted ineffective (data not shown). Since no other nuclear genetic markers have been developed so far for the study of the genetic structure of *Ae. koreicus*, SSR markers for this mosquito species have been developed in the present study.

### Methods

Putative SSR-containing sequences were identified in the DNA of *Ae. koreicus* thanks to the screening of raw sequencing data available in the Sequence Read Archive (SRA; Runs: ERR7598205–ERR7598207 [32, 33]) with the nucleotide Basic Local Alignment Search Tool (BLASTn), while primers for their amplification were designed using the web tool Primer3Plus [34, 35]. The amplification efficiency of each primer pair was tested with polymerase chain reaction (PCR). First, single PCRs were performed using DNA samples purified from individuals of *Ae. koreicus*. Primer pairs that proved to be effective were then labelled at the 5′ end with three different fluorophores (Table 1), and different combinations of multiplex PCRs were tested.

**Table 1:** Microsatellite sequences identified in the nuclear genome of *Aedes koreicus*, and organisation of the validated markers as multiplex PCR

	Locus name	Reference sequence (Run number.read number)	(Repeat motif)_n_^a^	Primer sequence (5′-3′)	PCR fluorophores^b^	Size range (bp)
Multiplex A	AK01	ERR7598207.14364316	(CAA)_7_	F: GCCGCTACAAACAGCTCAGT R: TCGTAAATTCGACGGGGTAG	TAMRA	121–127
	AK05	ERR7598207.14346661	(CAA)_6_	F: AACCGACTACGATGCACACA R: TAAACACAGAGCCAGCCACA	HEX	104–116
	AK07	ERR7598207.13874909	(GGC)_6_	F: ATCGAGCACACCGTCGTT R: ATCCATTTTTCTTTCTTTCGCTGT	6-FAM	144–150
	AK13	ERR7598207.13190639	(GTT)_6_	F:CCATTAAAAGGTTTTACTCAATGTTGT R: AAGAGACGGCTGTGGTCCT	TAMRA	141–144
Multiplex B	AK03	ERR7598207.14368852	(GAA)_6_	F: CGACTCCCTGAGCATAGTGT R: CATGCTATGATGAGCACATTGA	6-FAM	133–139
	AK09	ERR7598207.14366337	(ACG)_11_	F: TACCCGAATCCAGCAAACAT R: GCCAGAAGGACATCGTCAC	TAMRA	90–102
	AK10	ERR7598207.14369161	(ATC)_6_	F: AACTTGCCGCAATAGATGGT R: CCAAGACCTATTCTGGAAGCA	HEX	117–126
	AK12	ERR7598207.14357357	(ACA)_6_	F: CCAAAACGTATATCATCCGAAG R: GGAGAATCATCTGTGATAGTTTTGTG	6-FAM	91–97
Multiplex C	AK15	ERR7598207.14247464	(TAA)_7_	F: CCCAGTCCCAGCTTGAATAA R: AGGCATCAAGCTGCATCCTA	HEX	103–106
	AK23	ERR7598207.14317350	(CCTT)_5_	F: GGGGTTAACCAACCGAACC R: CGTTTGCCTTATACTGACAAATCT	6-FAM	134–145
	AK28	ERR7598207.14270387	(GGTT)_8_	F: GCAGCAACTTCCTTCCGTAG R: AACTGGCTGACCAGCGTAAC	6-FAM	80–104
Discarded	AK02	ERR7598207.1435939	(GCA)_10_	F: GGTGCGTCAGCAGCAGTAGT R: GGGATGAGAGACAGAGGGAAA		
	AK04	ERR7598207.14356098	(TGA)_8_	F: GATTAGATAATCCGGCATGGA R: TTAAGATCAACCAGTGGCATAAGA		
	AK06	ERR7598207.14366465	(CGT)_6_	F: CTCCGCCACCATTTTAGTGT R: TCCAGTTTTTCAAGAGCCATA		
	AK08	ERR7598207.12531158	(GGA)_7_	F: GCAGCCGGTAGTGGTGAC R: GCAGAATTGCTTGCTGCAC		
	AK11	ERR7598207.14345973	(AAT)_6_	F: TACCCGAATCCAGCAAACAT R: GCCAGAAGGACATCGTCAC		
	AK14	ERR7598207.14353635	(GAT)_8_	F: CGGCTTTGATGACAGGAAAC R: TGTTTACCAGACCGCACTGT		
	AK16	ERR7598207.14084369	(GAA)_6_	F: ACAATCCCTGGCAATGTCC R: CAGCCTGCACAAACAACAC		
	AK17	ERR7598207.14057111	(TCT)_6_	F: AGCAATGATGGGTGACTATTGTT R: AGCAATGATGGGTGACTATTGTT		
	AK18	ERR7598207.14365408	(GTC)_7_	F: CGATGTGGCCTTTTGTCG R: CTTCCAAAATTTCATAAAACACTGC		
	AK19	ERR7598207.14367708	(CA)_7_	F: CAACTGAAGAGTGAATTCCAAAA R: AGGGCGATACGGTCAAAAT		
	AK20	ERR7598207.8923957	(CG)_10_	F: GGTTTTCCCCGAGTTCGT R: GGGTGGGTGGGTTAATTTTC		
	AK21	ERR7598207.13745169	(AATT)_5_	F: GCTTTTTATCTGGTGAAATGCT R: AGCAGCAACACCATCATGC		
	AK22	ERR7598207.14102450	(GGAA)_5_	F: CGACTCGGTACGAGTTCACA R: CAACCGAACCAACCAATAGT		
	AK24	ERR7598207.14368656	(CT)_17_	F: GATTTCGAAACATGGTGAAAG R: GATGTAGCCATGATTGCAAGTAG		
	AK25	ERR7598207.14357143	(GA)_12_	F: AACTGTTCGCAATTGGCTTT R: ATTCATAGCACTCGGCGAAA		
	AK26	ERR7598207.14364132	(TG)_8_	F: CGCTCCGATTTTCGTATTCA R: CGTCGGGCTCAGACTATTTG		
	AK27	ERR7598207.14359268	(TA)_7_	F: GTCGGTGATTGTCACCATGT R: CATCCAGAGTGCATCAATCG		
	AK29	ERR7598206.10239627	(GGCC)_4_	F: CTACCCTTGCTTGGAGGTTG R: GTCGAGACGTGTGAGAGTGC		
	AK30	ERR7598207.11336441	(GC)_5_	F: TGAGTAACTGCGAGCTTGTCTC R: GAGATTGATTGTAAATACACACACACA		
	AK31	ERR7598207.14342765	(TGA)_6_	F: TCGCTGGAATGGTATAAGGAA R: TTGCCTTGCTACATTAGATGGT		

Indicated for each locus: the reference microsatellite-containing sequence (available in the Sequence Read Archive, SRA); the repeat motifs; forward (F) and reverse (R) primer sequences. In addition, for the 11 functional microsatellite markers, details about fluorophores used to mark primers, and the size ranges of the detected alleles (base pairs [bp]) are specified

^a^*n* = number of in tandem repeats of the microsatellite motive in the reference sequence

^b^Fluorophores were all added at 5′-forward primers, except for AK15, which has been marked on a 5′-reverse primer

The obtained microsatellite loci were assayed on a panel of 32 mosquito individuals collected in four different locations, representative of different areas of Northern Italy: the districts of Asti, Como, and Vicenza (collected in autumn 2021) and Bergamo (collected in summer 2022). Mosquitoes were identified as *Ae. koreicus* using the methodology described by Arnoldi et al. [14]. In particular, the DNA of eight mosquito samples from each location was extracted with the DNeasy Blood & Tissue kit (Qiagen, Hilden, Germany) and used in PCR. The PCR protocol that we developed for the single PCR is as follows. Final concentrations of PCR reagents in a 25 µl volume: 1 × Colorless GoTaq^®^ Reaction Buffer (Promega, Milan, Italy), 0.6 U GoTaq^®^ DNA polymerase (Promega, Milan, Italy), 0.2 mM dNTPs (Promega, Milan, Italy), 0.5 μM of each primer, and ~10 ng of DNA as template. Concerning multiplex PCRs, the final concentrations of the reagents is the same, except for primer pairs. Particularly, in multiplex A and B (both with four primer pairs), primer concentration was 0.125 μM; on the contrary, in multiplex C (three primer pairs), primer concentration was 0.167 μM (see Table 1). The thermal protocol for the amplification is identical for single and multiplex PCR reactions. The latter started with an initial denaturation at 94 °C for 2 min, followed by 40 cycles of denaturation at 94 °C for 30 s, primer annealing at 57 °C for 30 s and elongation at 72 °C for 30 s; a final extension of 5 min at 72 °C concluded the reaction.

The amplicons were visualised on 1.5% agarose gel, and only loci that showed clear single bands for all the amplified samples were considered for further testing (Additional File 1: Figure S1). Finally, as the last step of experimental validation, the precise length of the amplified fragments was determined, thus genotyping the examined mosquito individuals. Briefly, PCR products were directly sent to the laboratory of Eurofins Genomics—Europe Applied Genomics GmbH (Ebersberg, Germany) for fragment length analysis (FLA service). Amplicon lengths were determined by capillary electrophoresis with an ABI 3130 XL sequencing machine, using Promega ILS600 as standard. The SSR fragment data were analysed by the company using GeneMapper v.5 software (Applied Biosystem, Waltham, MA, USA). Before statistical analysis, all results were manually checked using Peak Scanner v.2 software (Applied Biosystem, Waltham, MA, USA), and allelic length rounded to the integer.

To obtain preliminary data about SSR polymorphism, the genotypes of all 32 individuals were analysed considering all mosquitoes as part of a unique population. Parameters of intra-population genetic diversity, such as number of alleles (Na), number of effective alleles (Ne), observed and expected heterozygosity (Ho and He, respectively), unbiased expected heterozygosity (uHe), Shannon diversity index (I), and inbreeding coefficient (F) were computed using GenAlEx 6.5 software [36]; in addition, Polymorphic Information Content (PIC) was computed using Cervus v. 3.0.7 software [37, 38].

## Results and discussion

Our initial in silico screening identified 31 putative SSR-containing sequences, named AK01–AK31 (see Table 1). All the loci showed perfect repetitions of di-, tri-, and tetranucleotides; specifically, the majority of the loci are trimeric, while AK19, AK20, AK24, AK25, AK26, AK27, and AK30 are characterised by dimeric motifs, and AK21, AK22, AK23, AK28, and AK29 are tetrameric.

Primers designed for SSR amplification were assayed in eight mosquito samples using PCR, and for 16 primers pairs the amplification was successful. After PCR validation, FLA of these 16 loci was performed on the 32 mosquito samples (Table 1). Most of the SSR electropherograms showed clearly distinguishable peaks, and the length of the fragments was in line with the expected range. However, two SSRs were discarded after this analysis: AK02 and AK24. Peaks of the AK02 locus were altered by the presence of stutters (both in *n* − 1 and *n* + 1 positions), and length was shorter than the expected size (observed: ~73 base pairs [bp]; expected: 101 bp). The size range of the AK24 locus was broad, and the presence of spurious peaks made correct peak calling impossible. Although the AK11, AK19, and AK21 loci showed no technical problems, they were not further considered because they were not polymorphic in the 32 samples analysed.

Primers for the amplification of the remaining 11 loci were combined in three multiplex PCRs (Table 1). Results from the analysis of the multiplexes were congruent with those of the electropherograms of the single loci (see Additional File 2: Figure S2 for some representative electropherograms of both single and multiplex reactions). This suggests that the combination of primer pairs does not affect the amplification efficiency of multiplexes, and this was true even among samples from different locations. Particularly, all individuals were successfully genotyped for most of the 11 loci. Missing values have been observed just in two samples: one mosquito from Asti at locus AK03, and another individual from Como in AK09.

The genotyping results that we obtained for the 32 individual mosquitoes were then used to evaluate the level of SSR variation in this test population sample (Table 2).

**Table 2:** Data of microsatellite variation in *Aedes koreicus* individuals

Locus	N	Na	Ne	Ho	He	uHe	I	F	PIC
AK01	32	3	1.427	0.219	0.299	0.304	0.577	0.269	0.279
AK03	31	3	2.424	0.226	0.587	0.597	0.960	0.616	0.502
AK05	32	4	2.395	0.594	0.583	0.592	1.064	−0.019	0.525
AK07	32	3	1.683	0.313	0.406	0.412	0.734	0.230	0.370
AK09	31	6	3.161	0.548	0.684	0.695	1.344	0.198	0.636
AK10	32	4	1.416	0.250	0.294	0.299	0.562	0.150	0.266
AK12	32	3	2.002	0.563	0.500	0.508	0.748	−0.124	0.390
AK13	32	2	1.032	0.031	0.031	0.031	0.080	−0.016	0.030
AK15	32	2	1.032	0.031	0.031	0.031	0.080	−0.016	0.030
AK23	32	3	1.722	0.406	0.419	0.426	0.740	0.031	0.375
AK28	32	6	3.303	0.813	0.697	0.708	1.385	−0.165	0.645
Mean	31.818	3.545	1.963	0.363	0.412	0.419	0.752	0.105	0.368
SE	0.122	0.413	0.235	0.074	0.070	0.071	0.130	0.067	0.209

The following parameter are reported: number of individuals (*N*), number of alleles (Na), number of effective alleles (Ne), observed (Ho) and expected (He) heterozygosities, unbiased expected heterozygosity (uHe), Shannon diversity index (I), inbreeding coefficient (F), Polymorphic Information Content (PIC)

The most variable loci were AK09 and AK28. These displayed the highest allelic abundance (Na = 6 for both; Table 2), together with the maximum values of He and PIC (AK09: He = 0.684, PIC = 0.636; AK28: He = 0.697, PIC = 0.645) (Table 2). Even if AK03 and AK05 showed a limited number of alleles (Na = 3 and 4, respectively), the associated PIC values are higher than the average (mean PIC = 0.368 ± 0.209; PIC_AK03_ = 0.502, PIC_AK05_ = 0.525) (Table 2).

## Conclusions

In conclusion, we report the identification of microsatellite markers in the *Ae. koreicus* mosquito, the protocol for their amplification, and the preliminary genetic results on a limited number of individuals. The SSRs here implemented showed a good level of polymorphism. In addition to the novelty of designing molecular markers specific for the Korean bush mosquito, the SSRs here proposed will likely represent a useful tool for generating data and drawing hypotheses about the original introduction of *Ae. koreicus* in Europe, reconstructing its invasive routes and thus having more information to prevent the further spreading of the species.

### Supplementary Information

**Additional file 1: Figure S1 **Agarose gel electrophoresis showing the amplification of all 31 microsatellite sequences identified in the genome of *Aedes koreicus*. For all the loci (11 validated markers and the discarded ones), four representative DNA samples of *Ae. koreicus *were amplified with primer pairs designed in this study. **a** Pictures of amplicons obtained with AK01–AK10. **b** Pictures of amplicons obtained with AK11–AK20. **c**. Pictures of amplicons obtained with AK21–AK11. *M* molecular marker (GeneRuler 100 bp Plus DNA Ladder, Thermo Scientific™, Waltham, MA, USA); *A* Adult mosquito of *Ae. Koreicus*, *N* negative control.

**Additional file 2: Figure S2 **Representative electropherograms of single and multiplex PCR performed to validate the efficiency of the 11 microsatellite markers identified in the genome of *Aedes koreicus*; electropherograms were visualised with Peak Scanner v.2 (Applied Biosystems, Waltham, MA, USA). **a–k** Peaks associated with a single marker (one page for each locus). **l–n** Multiplex A, B, and C are reported (one page for all the loci involved in the same multiplex assay). The legend displaying the dye colours, and the corresponding fluorophores/loci, together with the distribution of the microsatellite markers in the multiplex assays, are reported at the end of the document.

## Data Availability

All data supporting the findings of this study are available within the paper and its Supplementary Information. Microsatellite primer sequences and alleles details are provided in Table 1; agarose gel electrophoresis pictures and representative electropherograms peaks are reported in Additional File 1 and Additional File 2, respectively.

## Abbreviations

SSRs: Simple sequence repeats
SRA: Sequence Read Archive
BLAST: Basic Local Alignment Search Tool
PCR: Polymerase chain reaction
FLA: Fragment length analysis
