# Supplementary figures and images for: Development of microsatellite markers for the invasive mosquito Aedes koreicus
Source: Parasit Vectors. 2023 Jul 6;16:223. doi: 10.1186/s13071-023-05823-z (PMC10324130; doi:10.1186/s13071-023-05823-z)

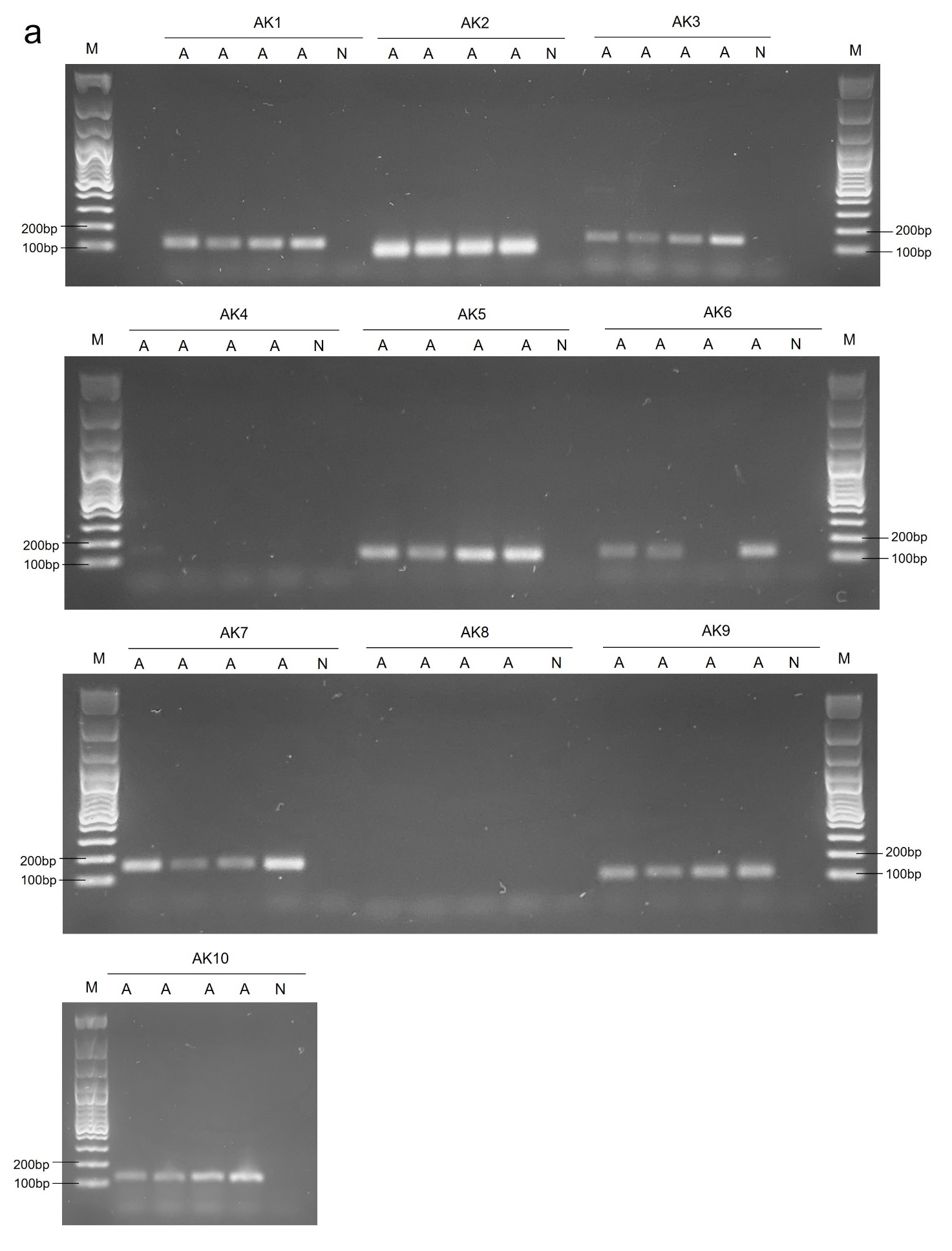


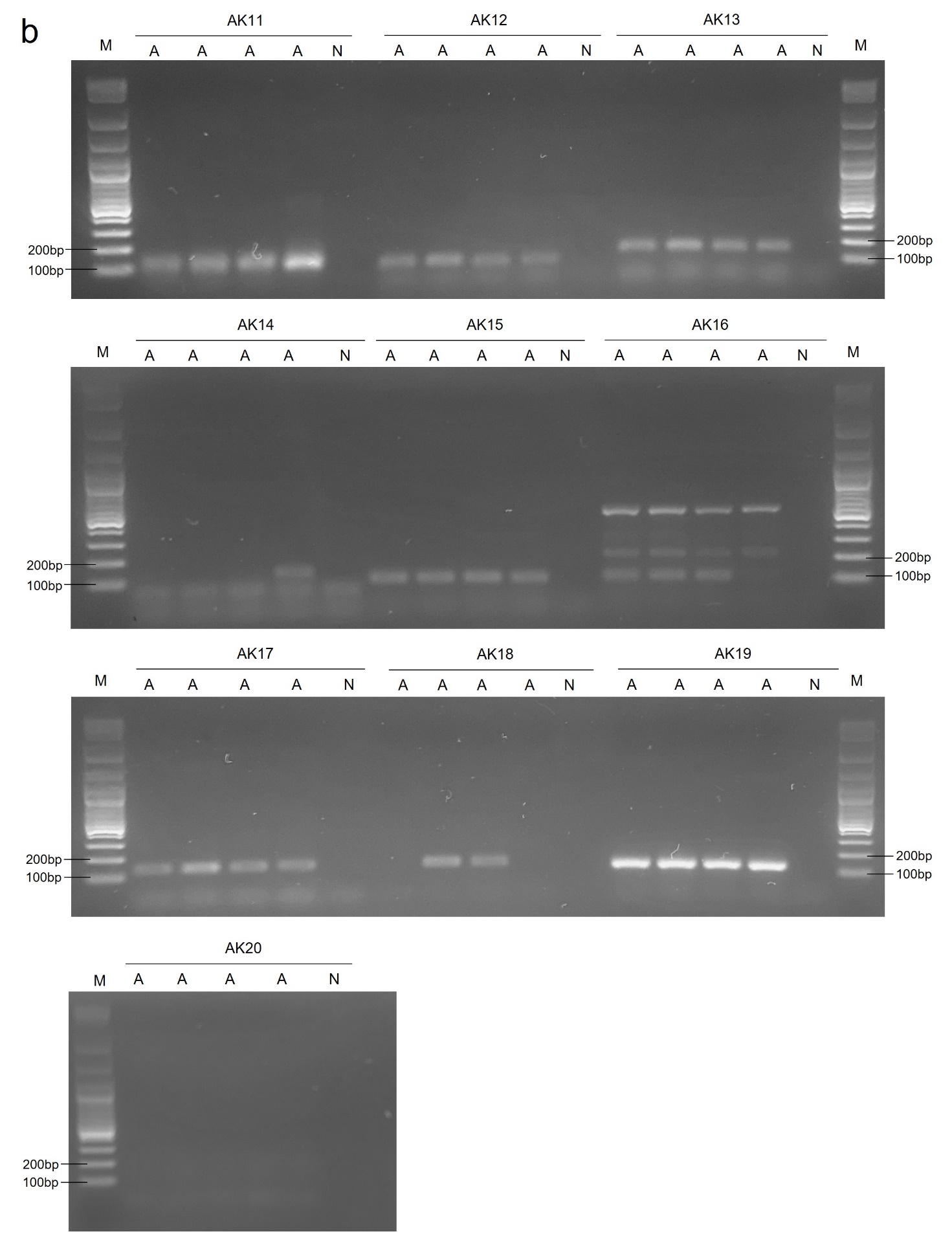

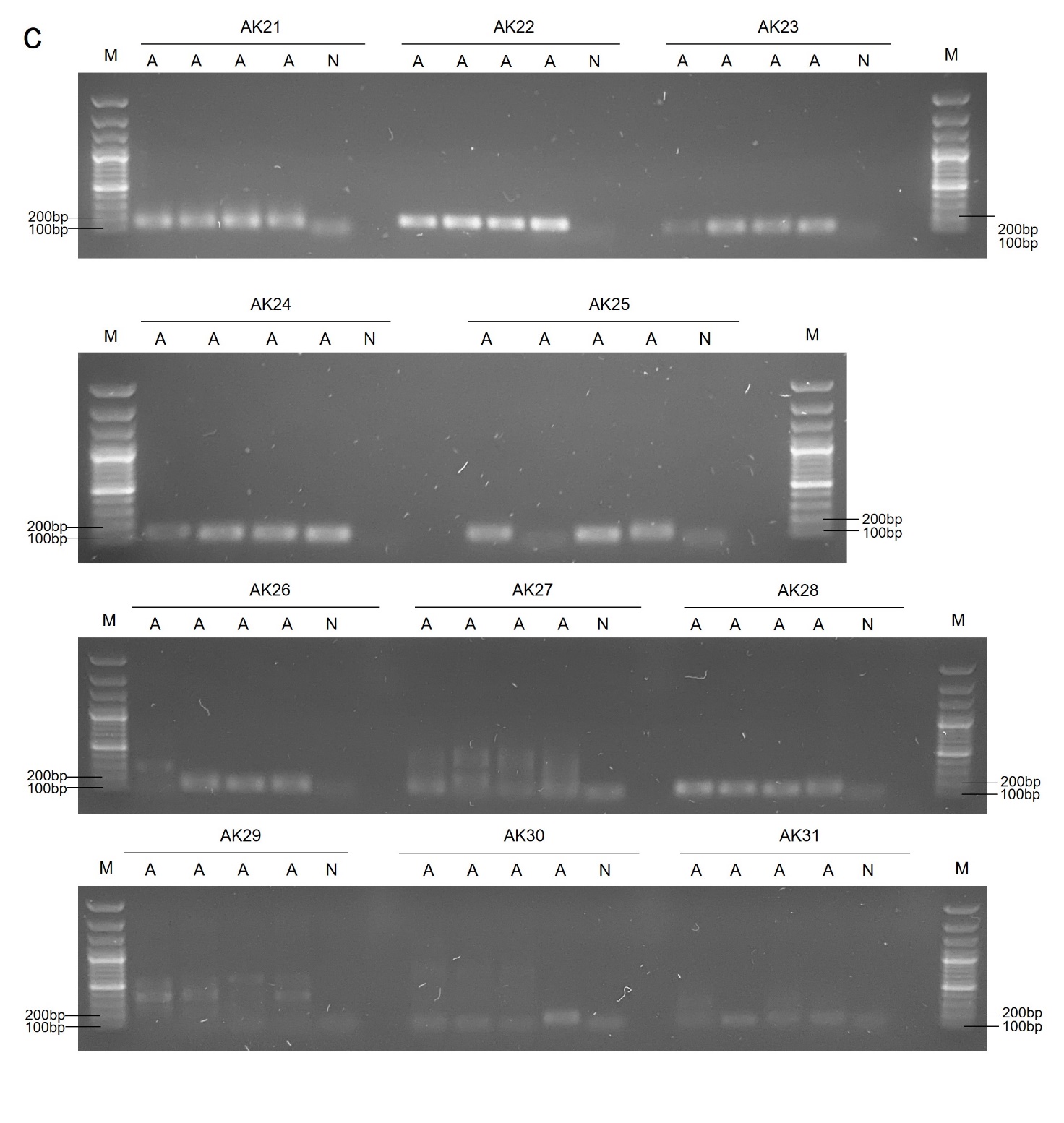

Supplement: Supplementary file 1 — Additional file 1: Figure S1 Agarose gel electrophoresis showing the amplification of all 31 microsatellite sequences identified in the genome of Aedes koreicus. For all the loci (11 validated markers and the discarded ones), four representative DNA samples of Ae. koreicus were amplified with primer pairs designed in this study. a Pictures of amplicons obtained with AK01–AK10. b Pictures of amplicons obtained with AK11–AK20. c. Pictures of amplicons obtained with AK21–AK11. M molecular marker (GeneRuler 100 bp Plus DNA Ladder, Thermo Scientific™, Waltham, MA, USA); A Adult mosquito of Ae. Koreicus, N negative control. [file 13071_2023_5823_MOESM1_ESM.docx]
