# Supplementary material for: Development of microsatellite markers for the invasive mosquito Aedes koreicus
Source: Parasit Vectors. 2023 Jul 6;16:223. doi: 10.1186/s13071-023-05823-z (PMC10324130; doi:10.1186/s13071-023-05823-z)
Supplement: Supplementary file 2 — Additional file 2: Figure S2 Representative electropherograms of single and multiplex PCR performed to validate the efficiency of the 11 microsatellite markers identified in the genome of Aedes koreicus; electropherograms were visualised with Peak Scanner v.2 (Applied Biosystems, Waltham, MA, USA). a–k Peaks associated with a single marker (one page for each locus). l–n Multiplex A, B, and C are reported (one page for all the loci involved in the same multiplex assay). The legend displaying the dye colours, and the corresponding fluorophores/loci, together with the distribution of the microsatellite markers in the multiplex assays, are reported at the end of the document. [file 13071_2023_5823_MOESM2_ESM.pdf]

a

1 A10_AK1_FonA-v50_APG22_PR0954.fsa

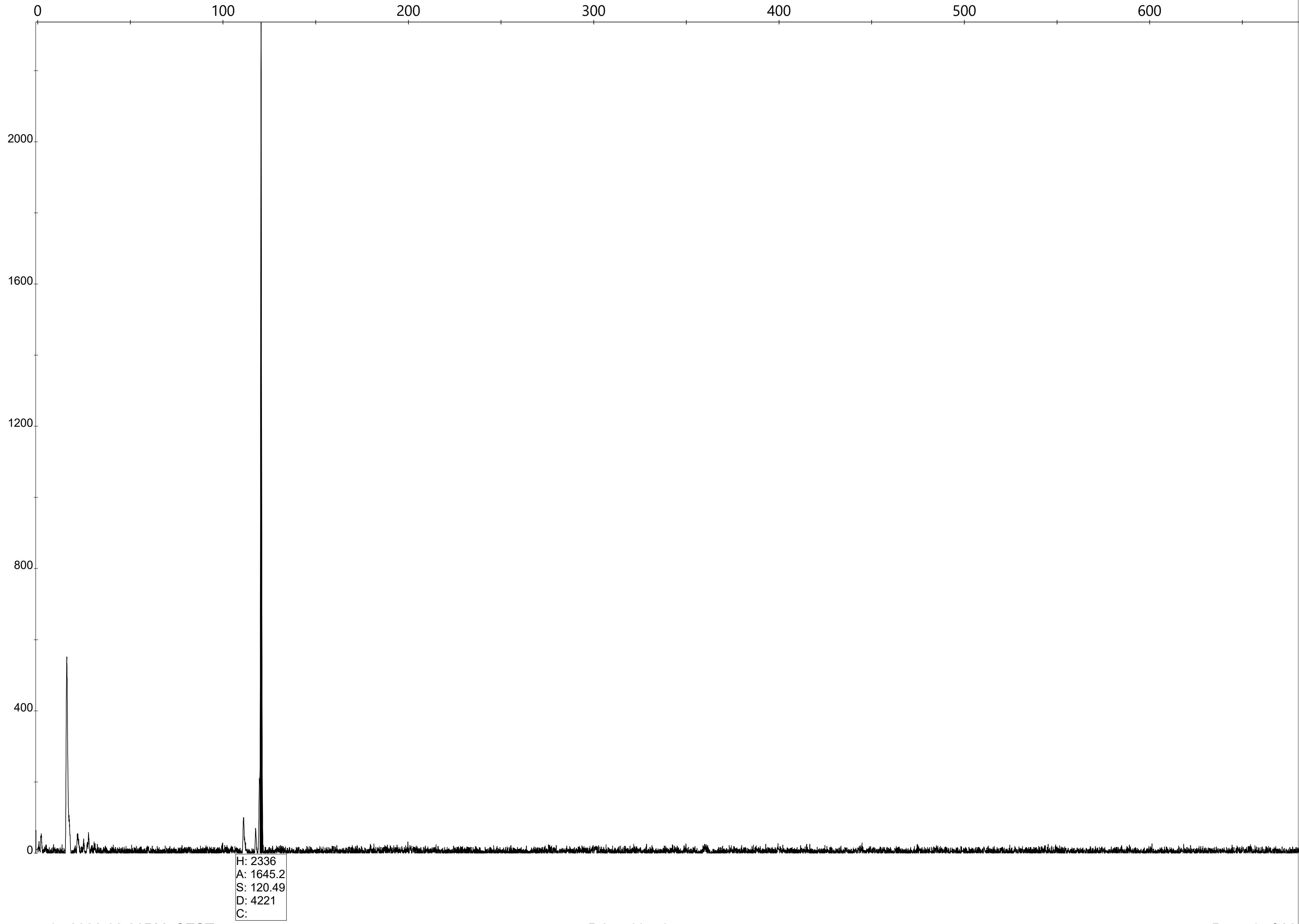

2 D03_AK3_FonB-v20_APG22_PR0983.fsa

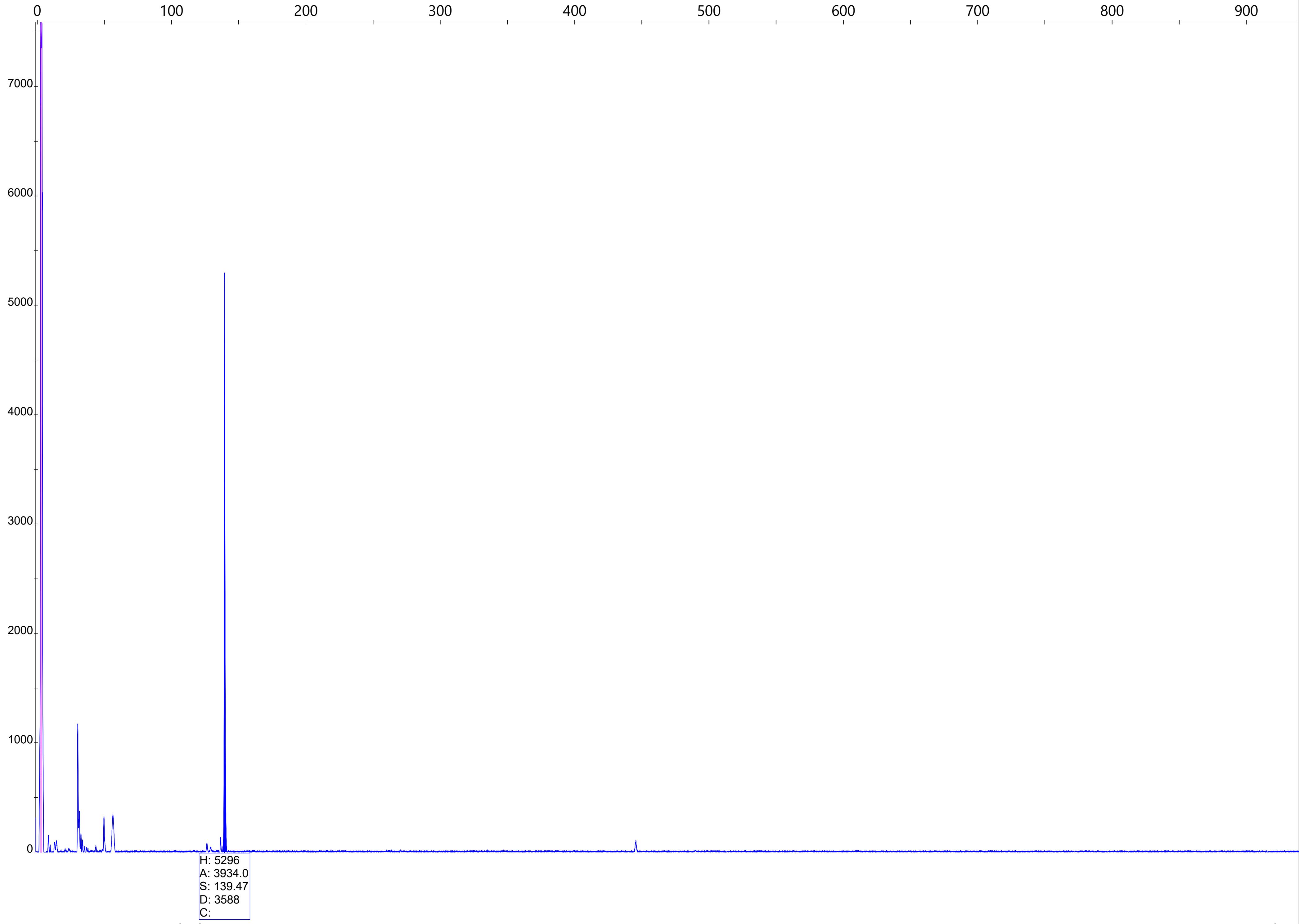

3 C10_AK5_FonA-v50_APG22_PR0954.fsa

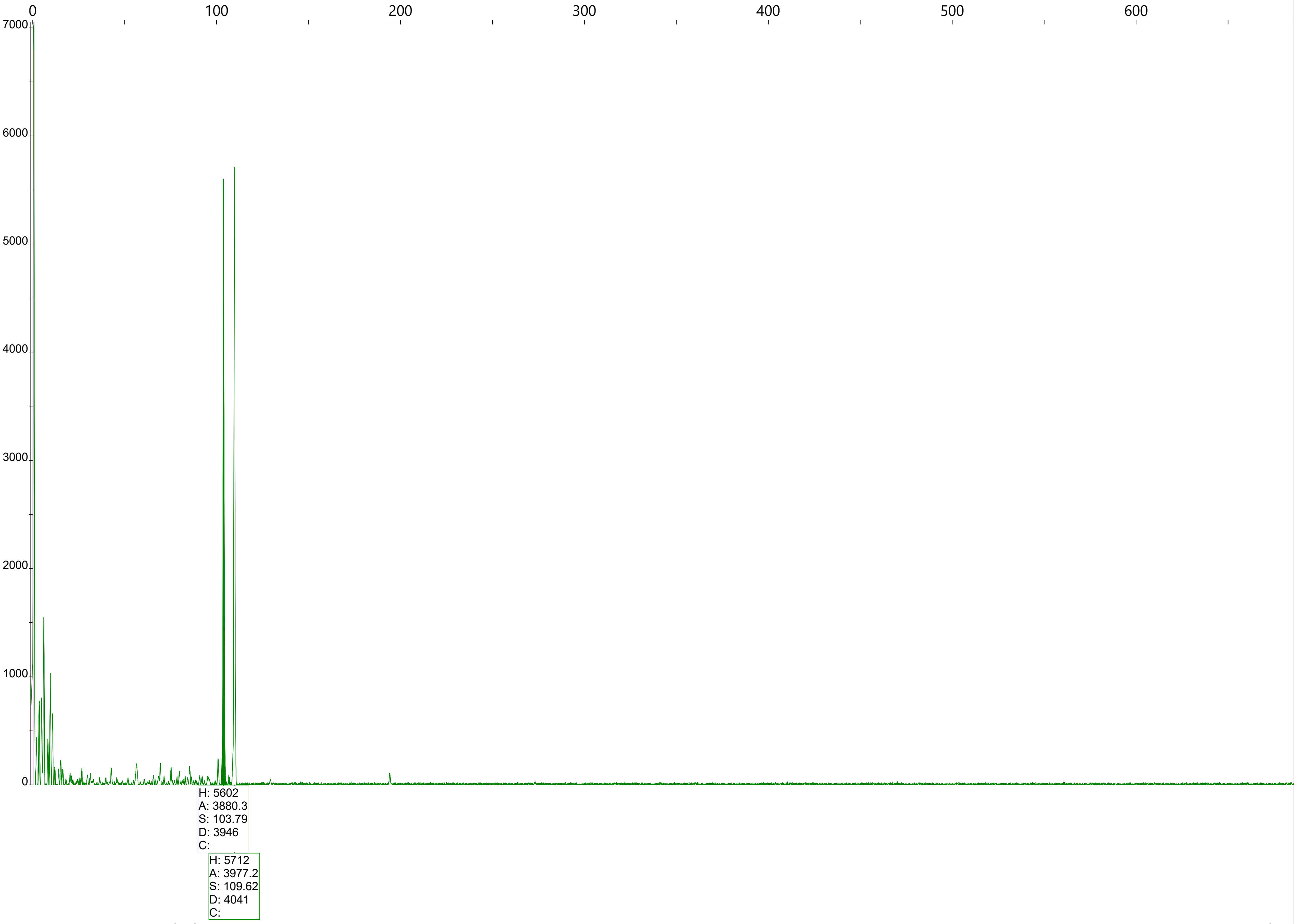

4 E10_AK7_FonA-v50_APG22_PR0954.fsa

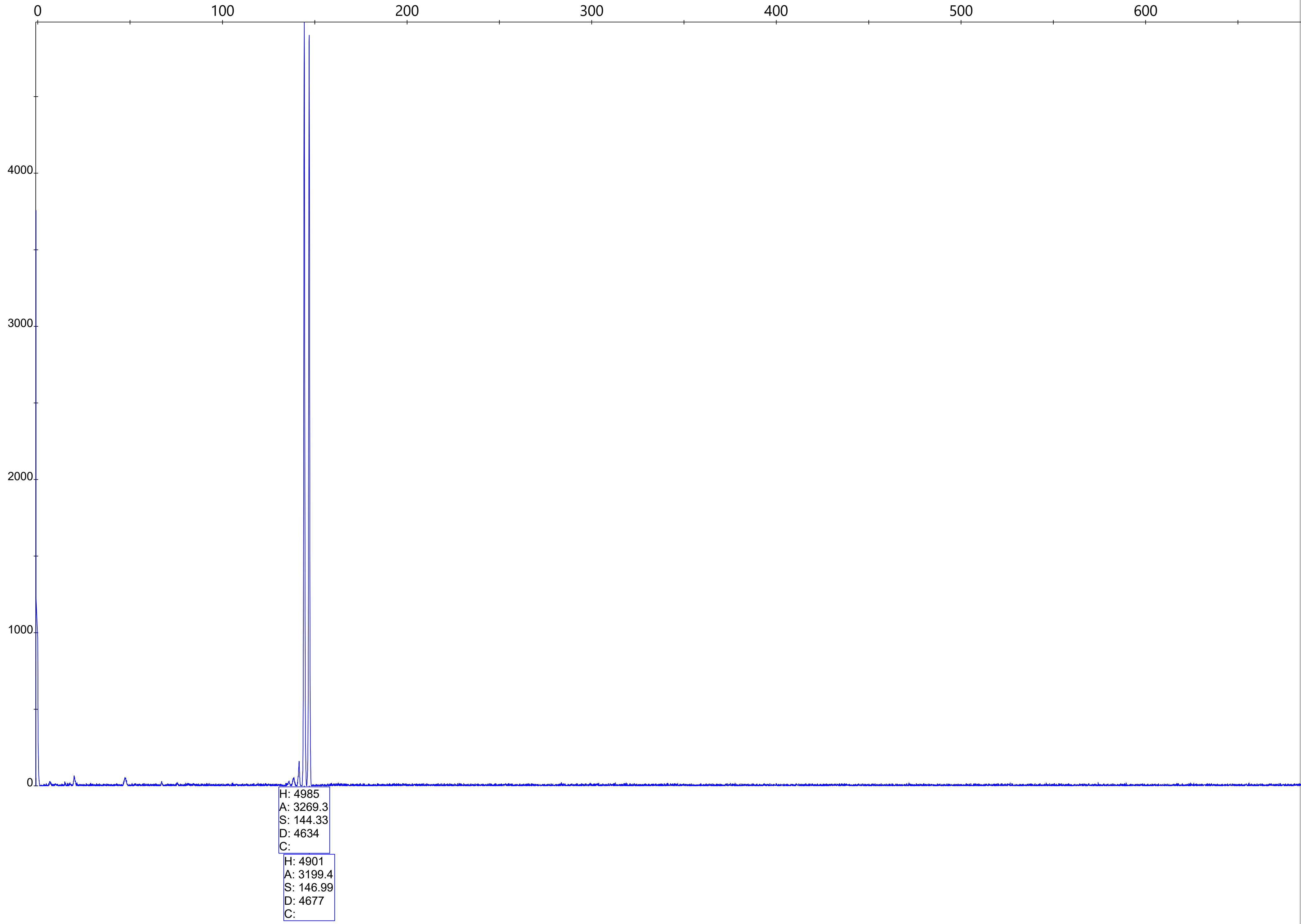

5 G10_AK9_FonA-v50_APG22_PR0954.fsa

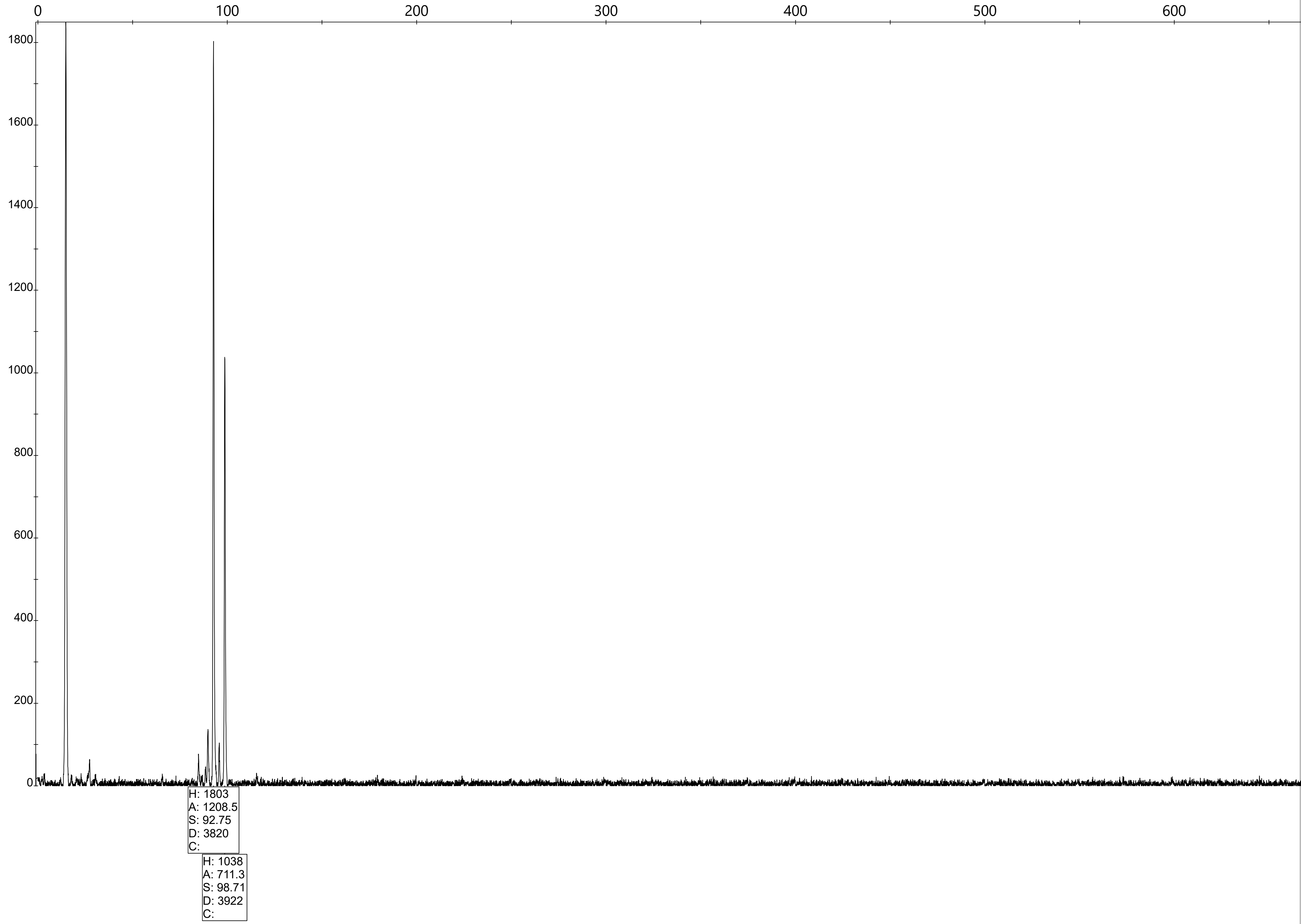

6 E03_AK10_FonA-v20_APG22_PR0983.fsa

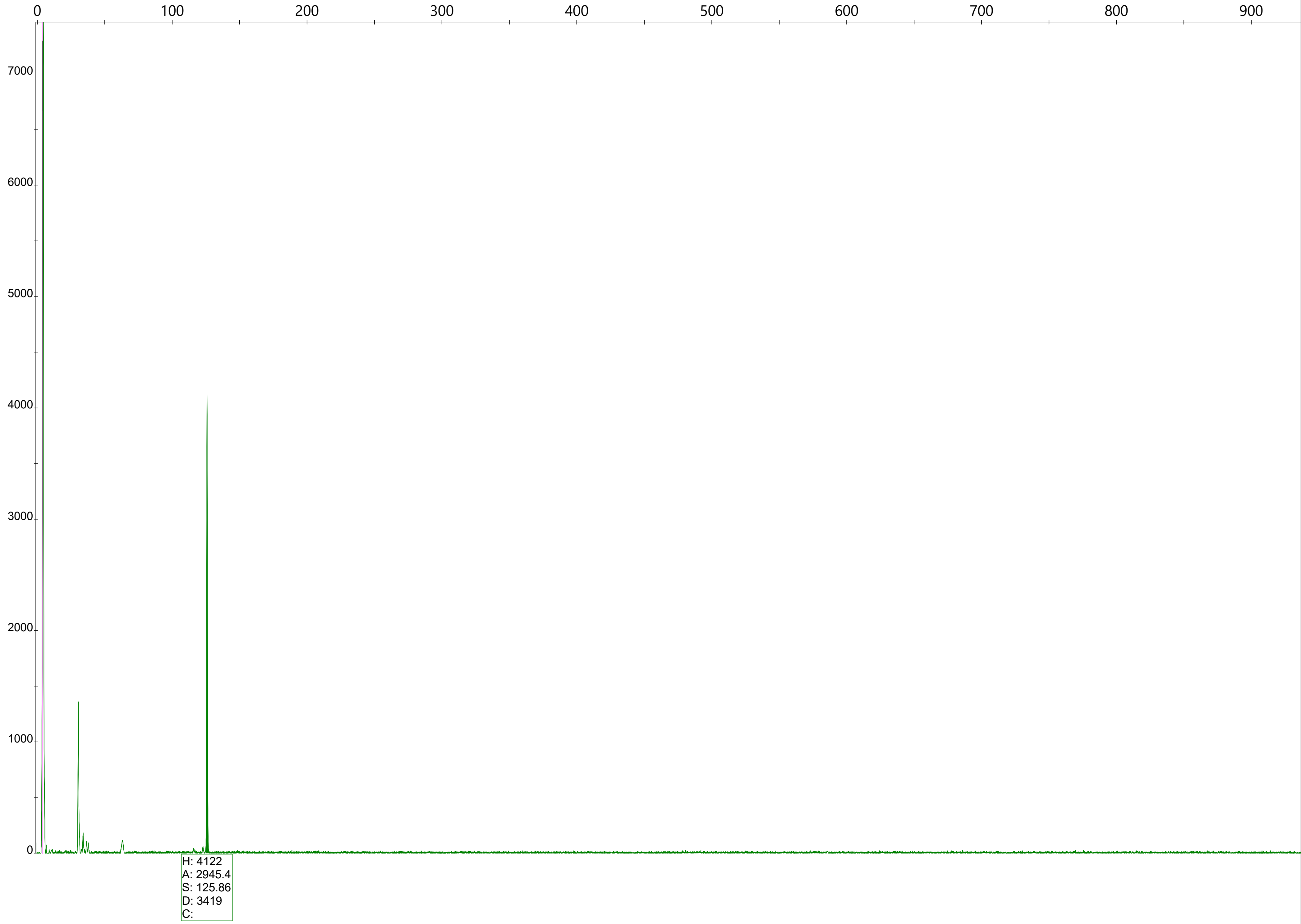

7 A02_AK12_FonA-v50_APG22_PR0983.fsa

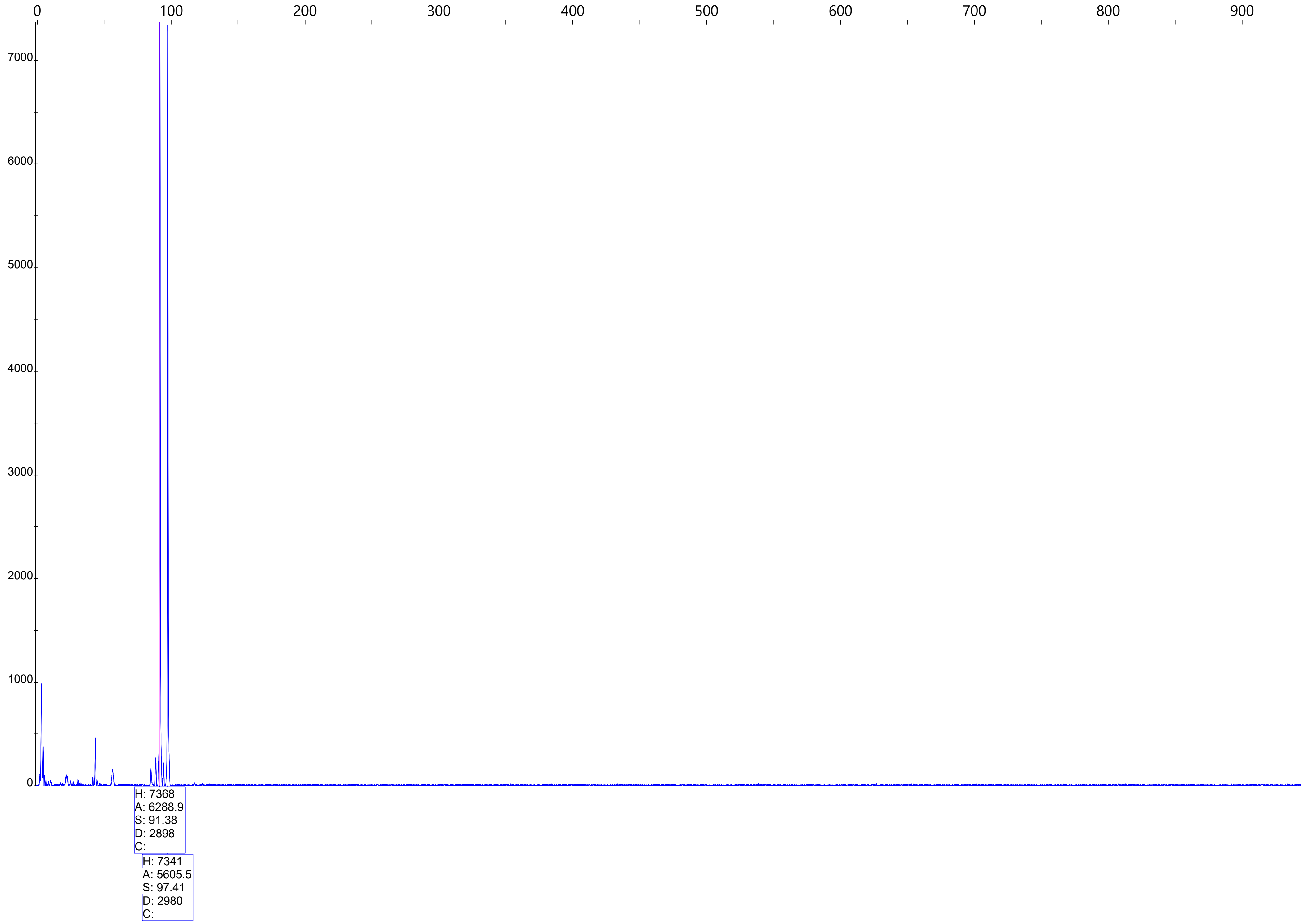

8 C02_AK13_FonA-v50_APG22_PR0983.fsa

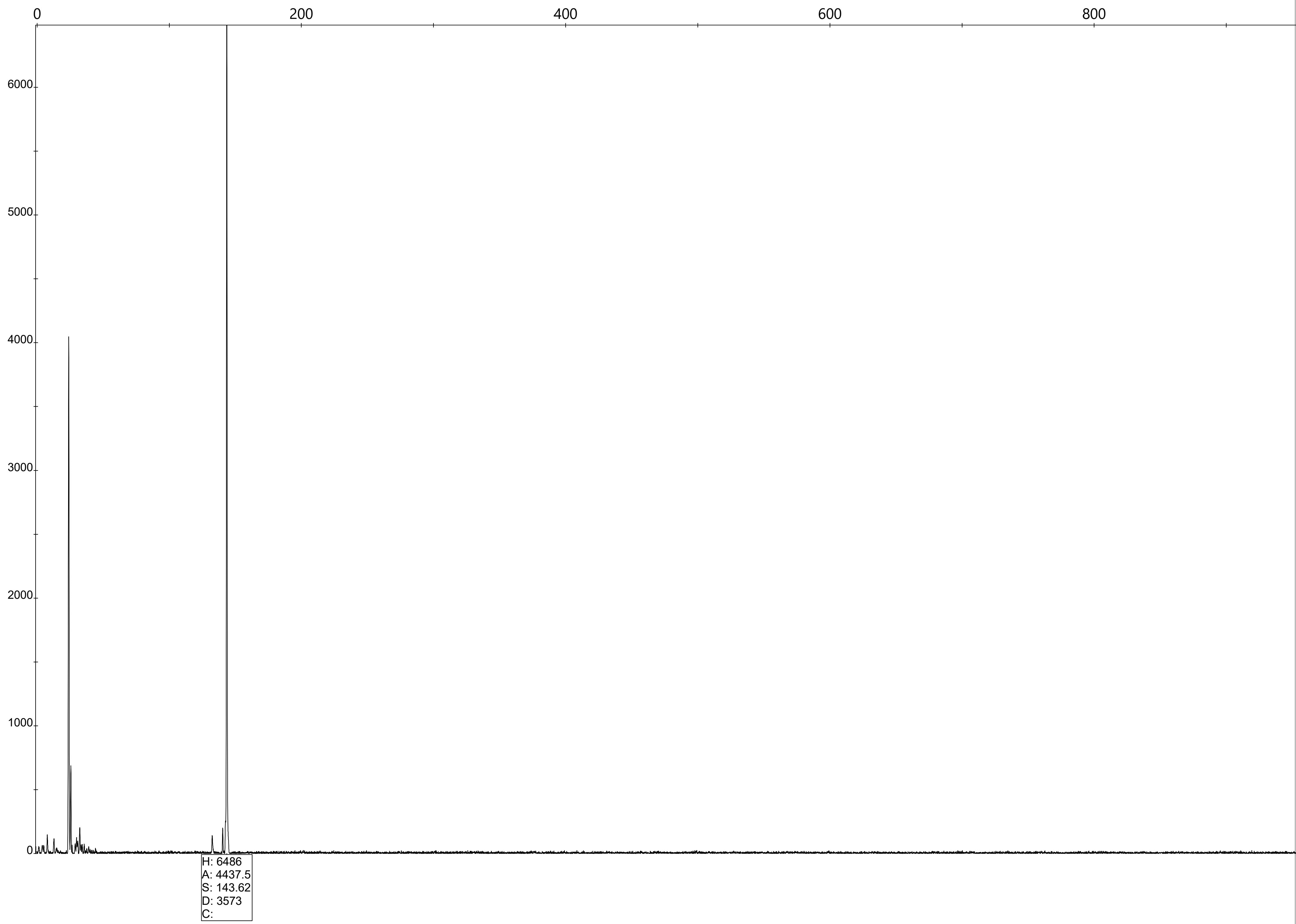

9 F11_AK15_FO2_APG23_PR0332.fsa

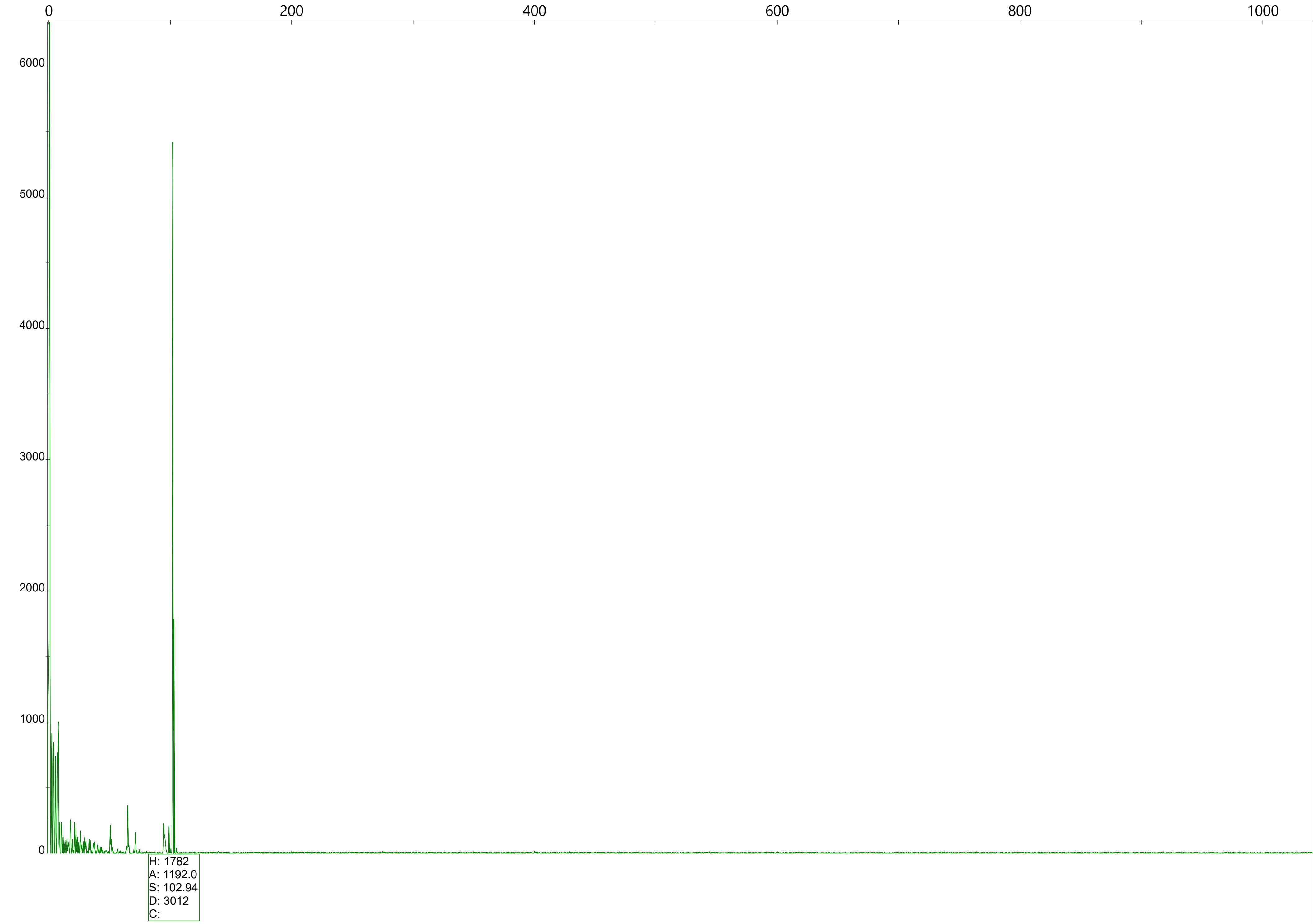

10 C10_AK23_FO1_APG22_PR1339.fsa

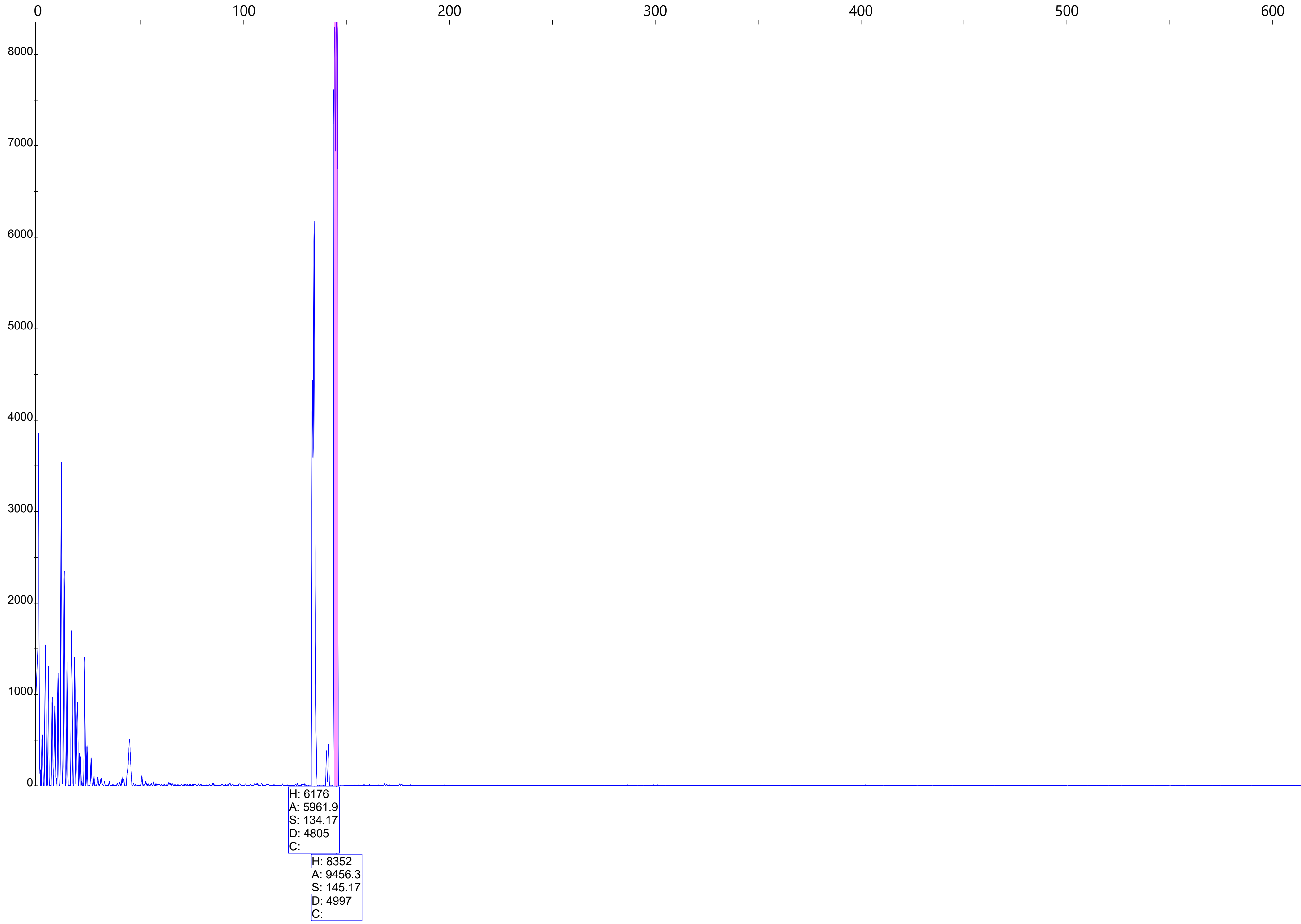

11 D11_AK28_FO4_APG22_PR1651.fsa

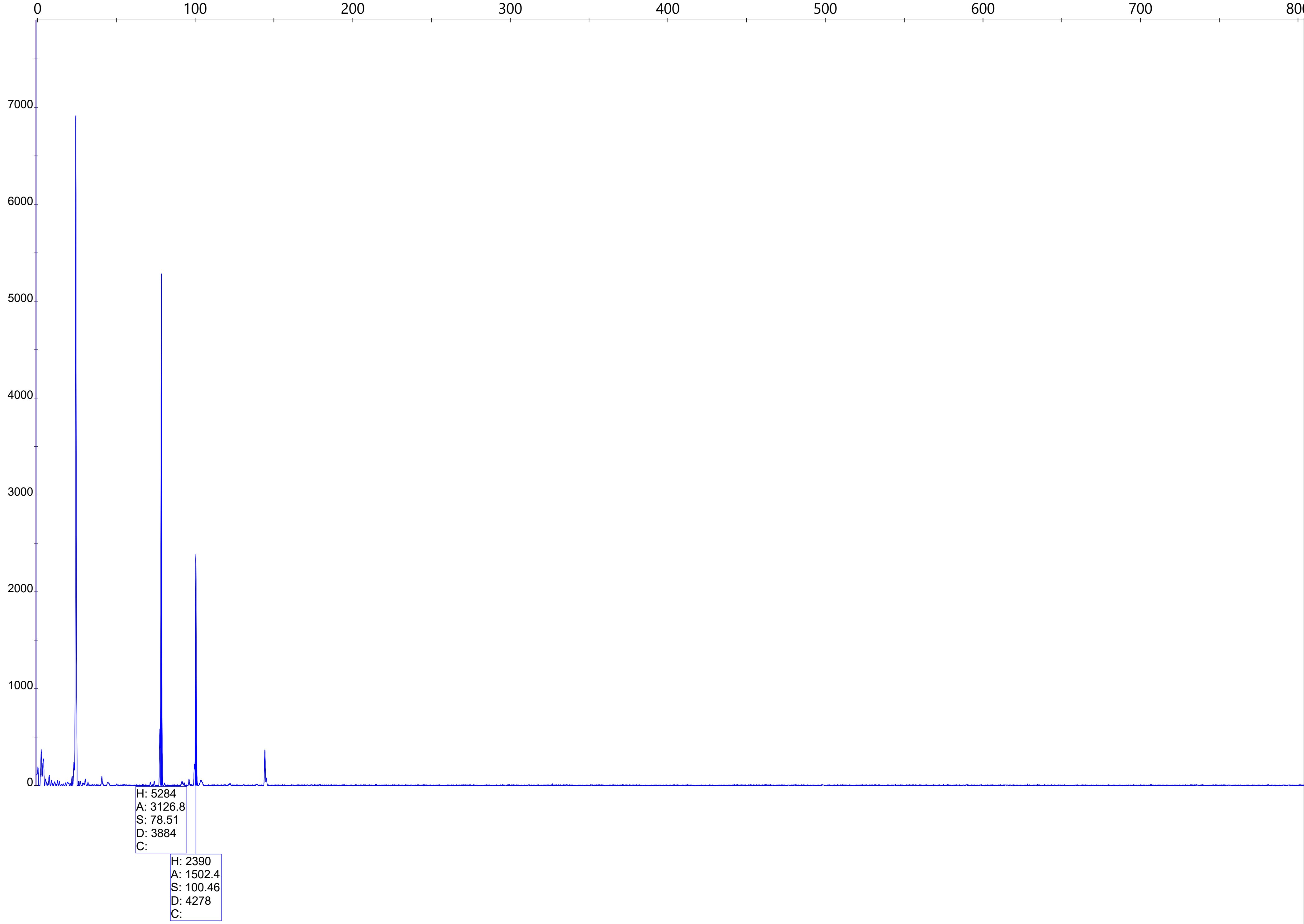

45 A01_MultiA_FO8_APG22_PR1426.fsa

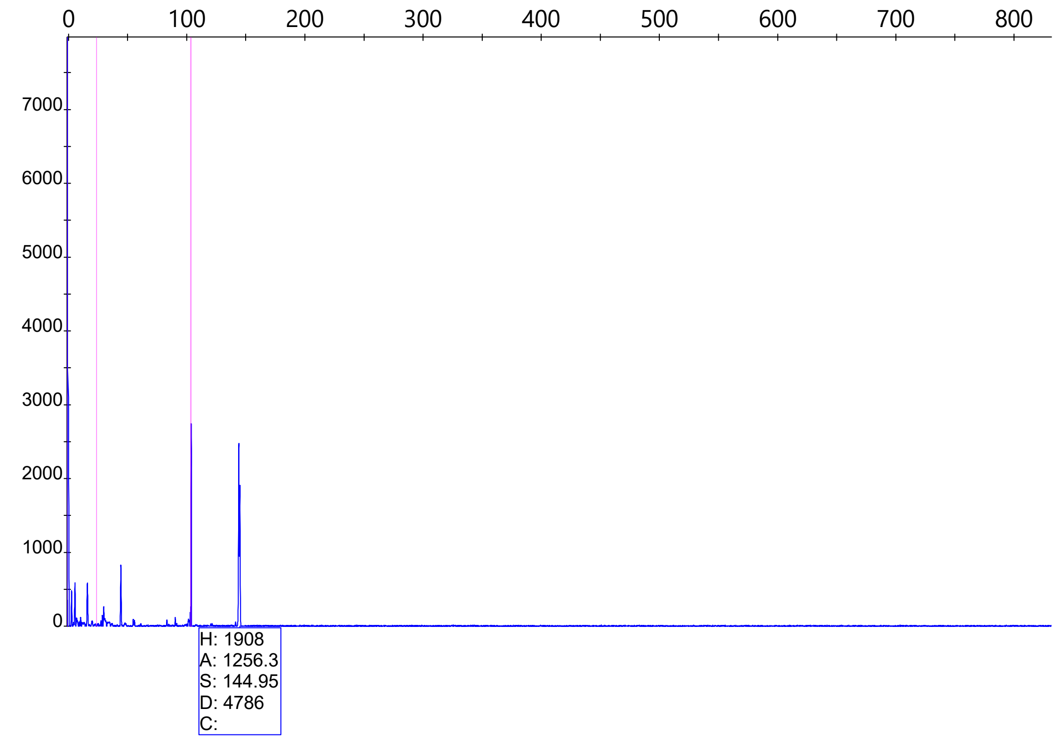

46 A01_MultiA_FO8_APG22_PR1426.fsa

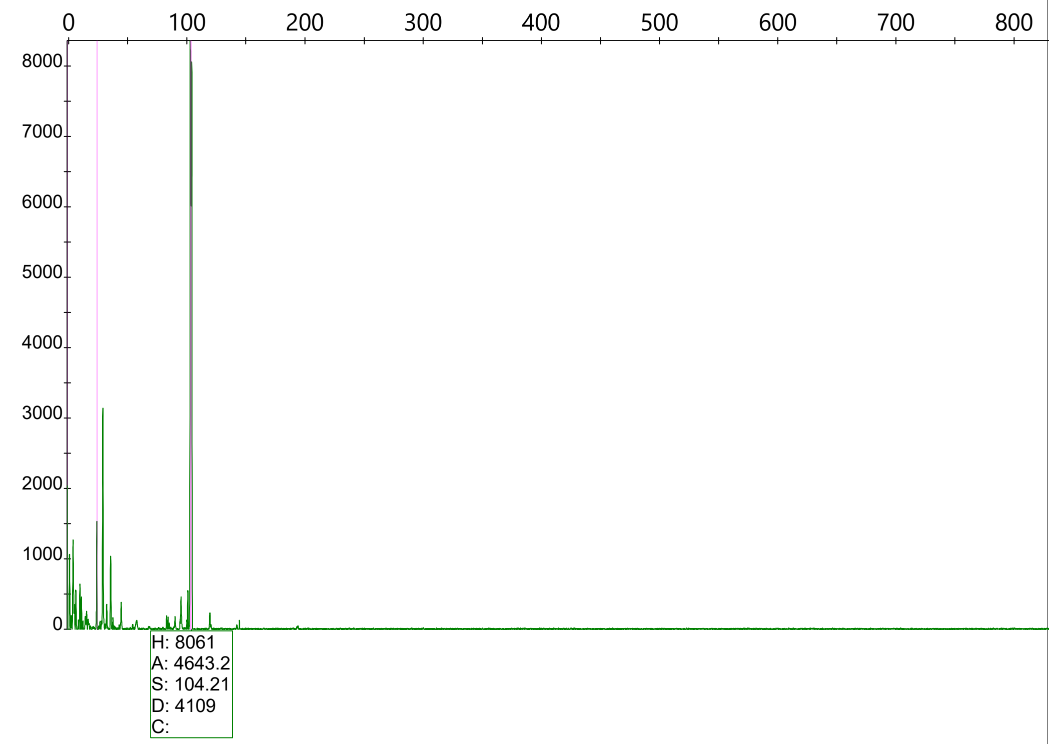

47 A01_MultiA_FO8_APG22_PR1426.fsa

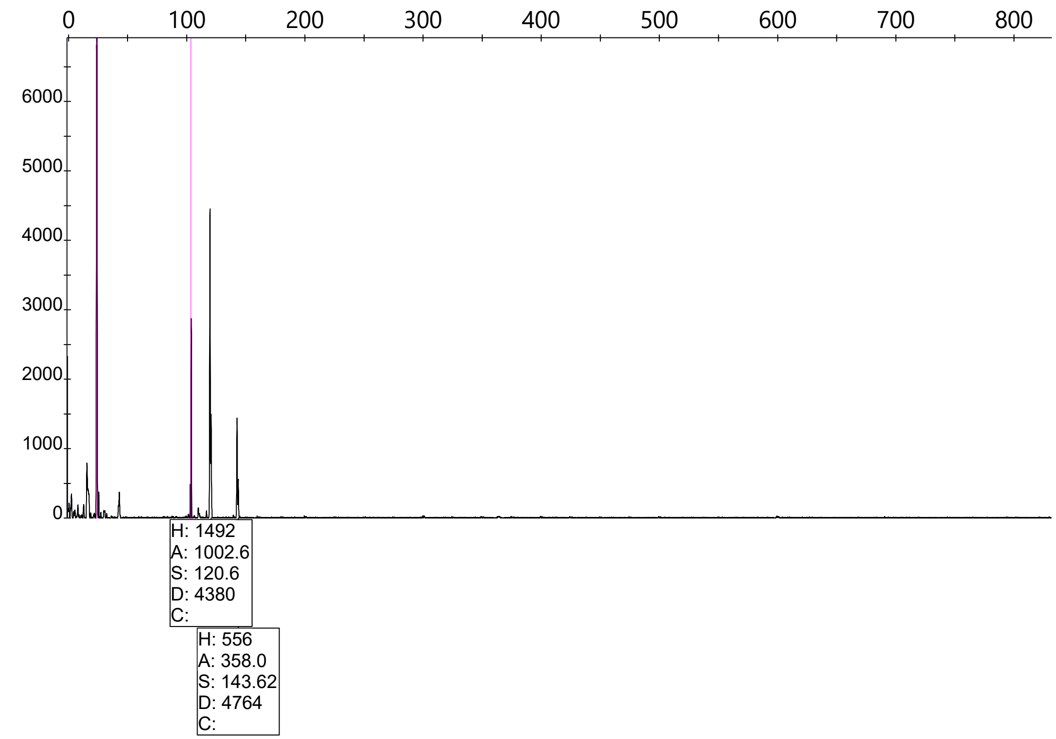

48 A01_MultiA_FO8_APG22_PR1426.fsa

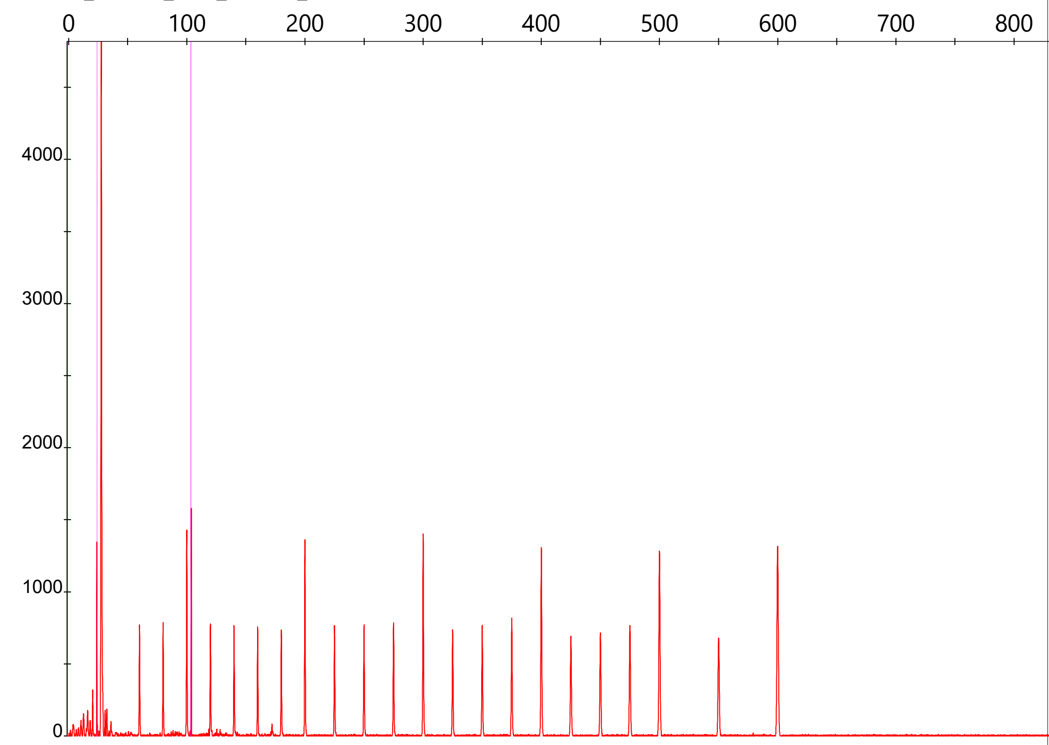

49 D12_MultiB_FO6_APG22_PR1651.fsa

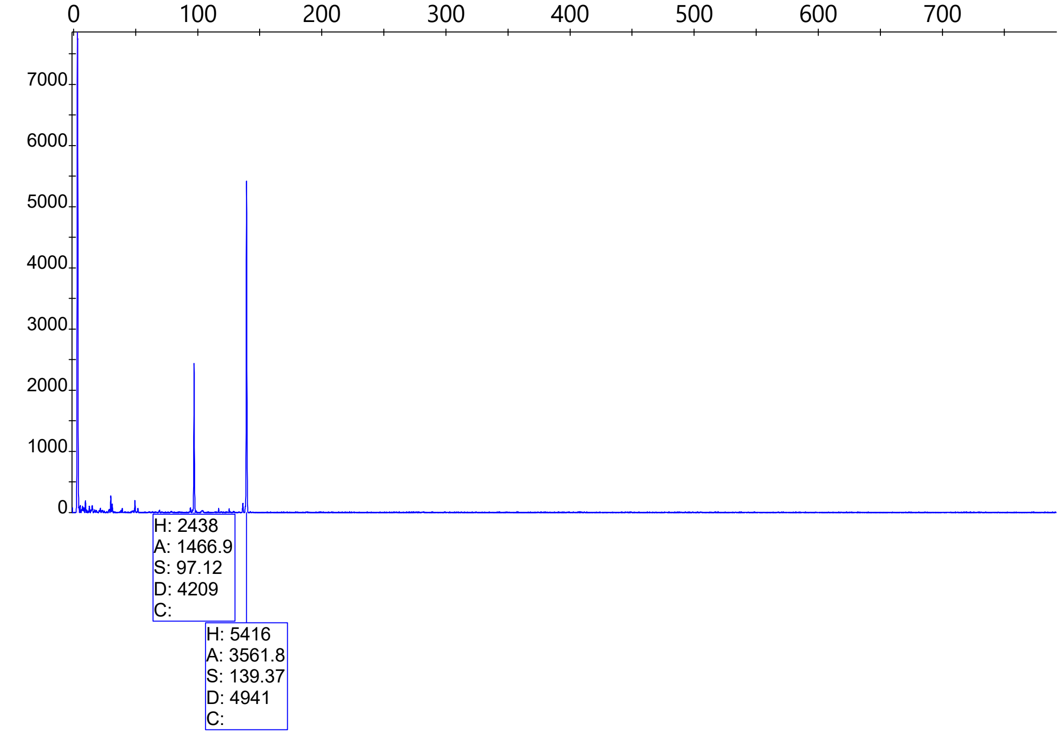

50 D12_MultiB_FO6_APG22_PR1651.fsa

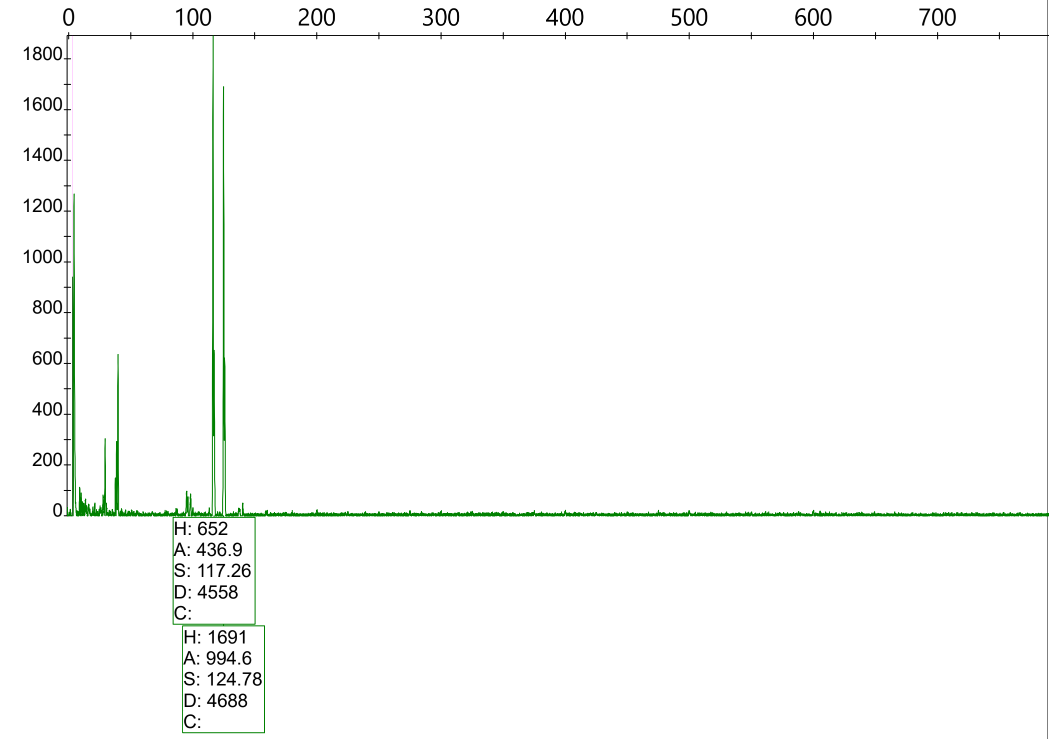

51 D12_MultiB_FO6_APG22_PR1651.fsa

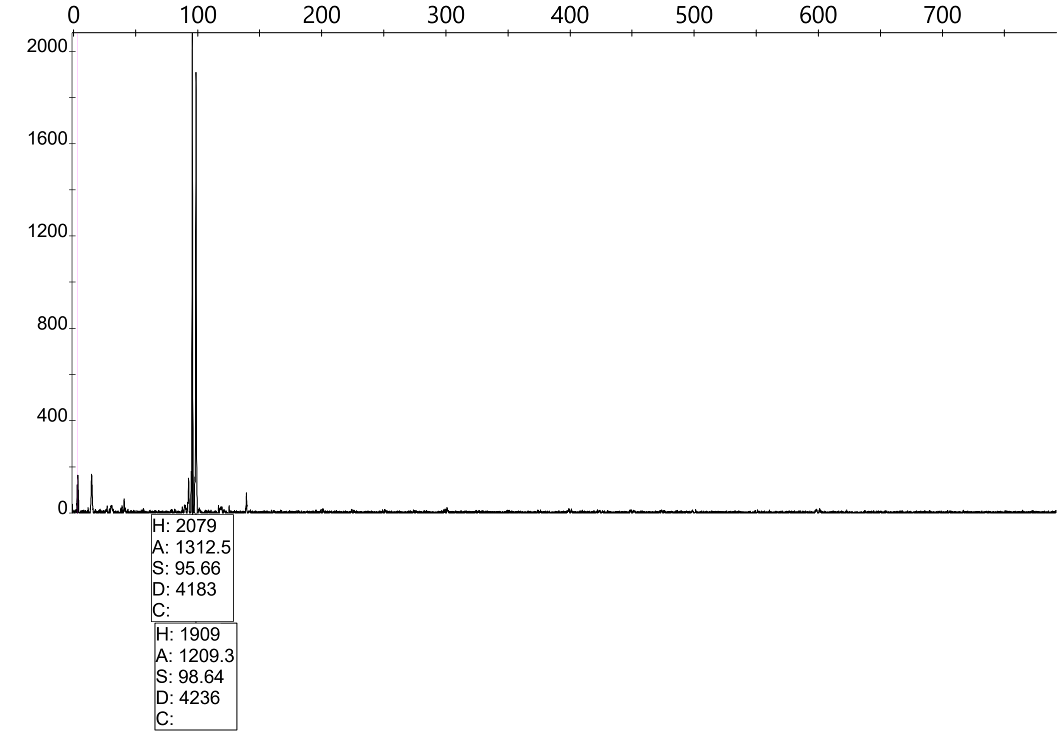

52 D12_MultiB_FO6_APG22_PR1651.fsa

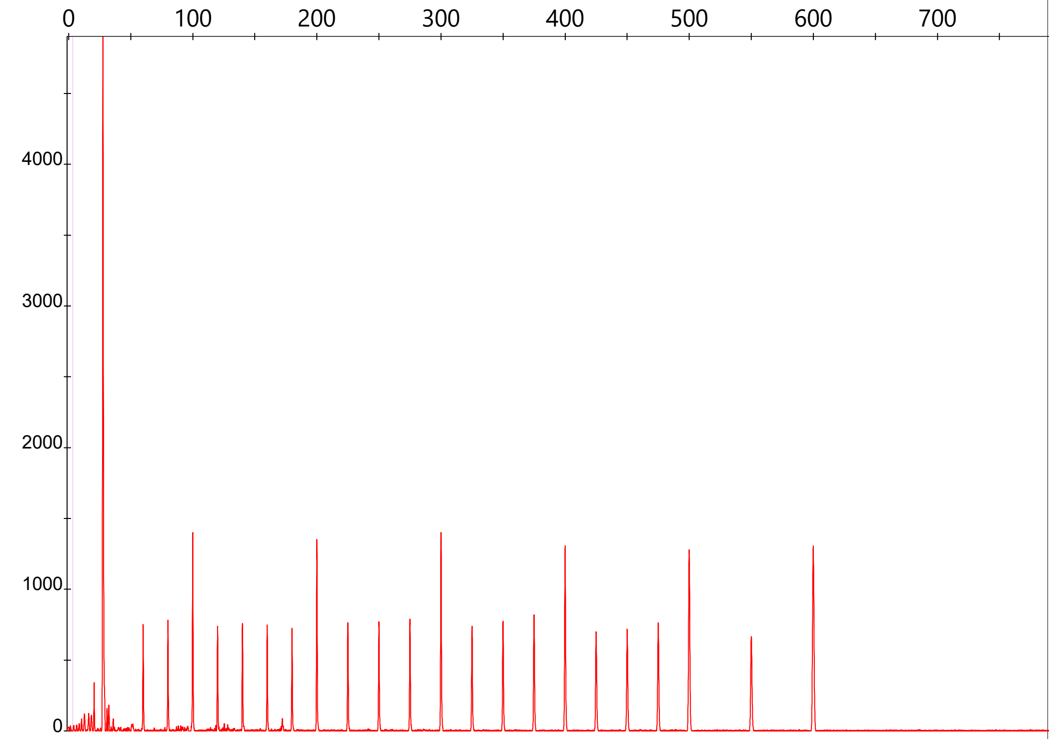

53 A10_MultiC_FO3_APG23_PR0643.fsa

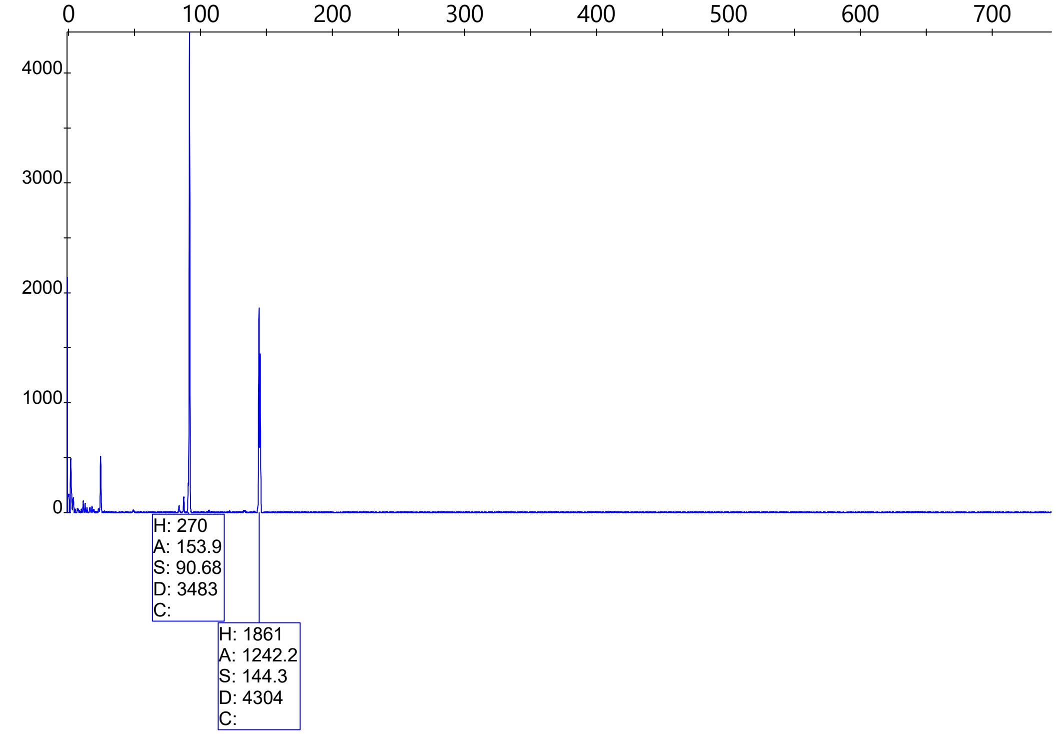

54 A10_MultiC_FO3_APG23_PR0643.fsa

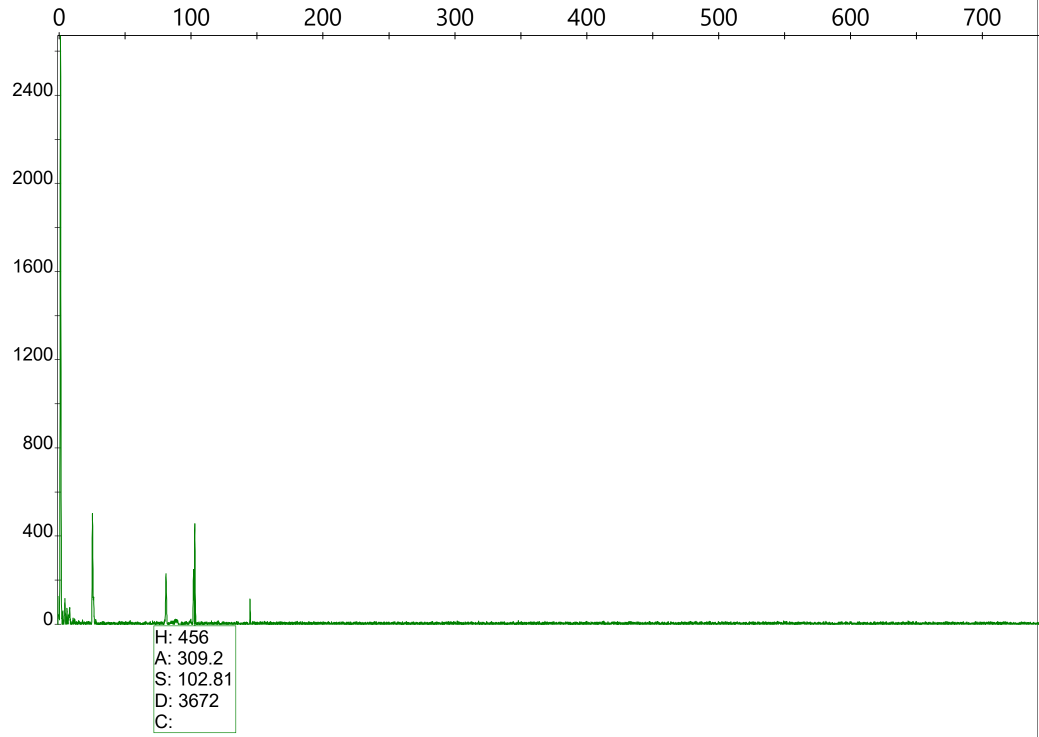

55 A10_MultiC_FO3_APG23_PR0643.fsa

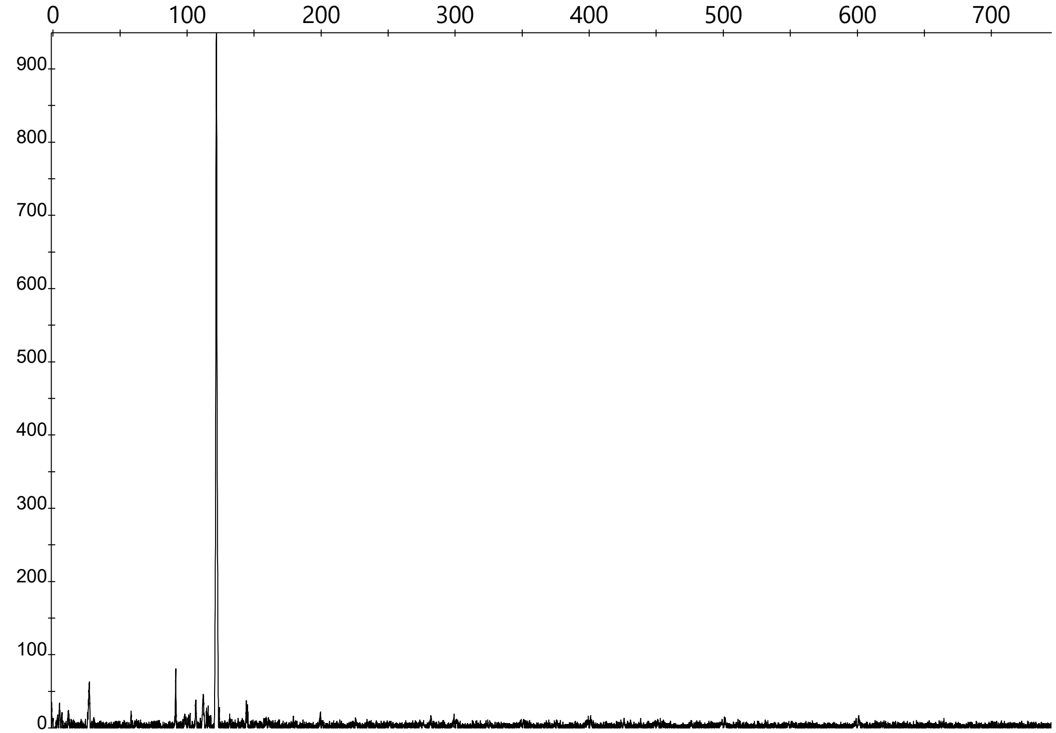

56 A10_MultiC_FO3_APG23_PR0643.fsa

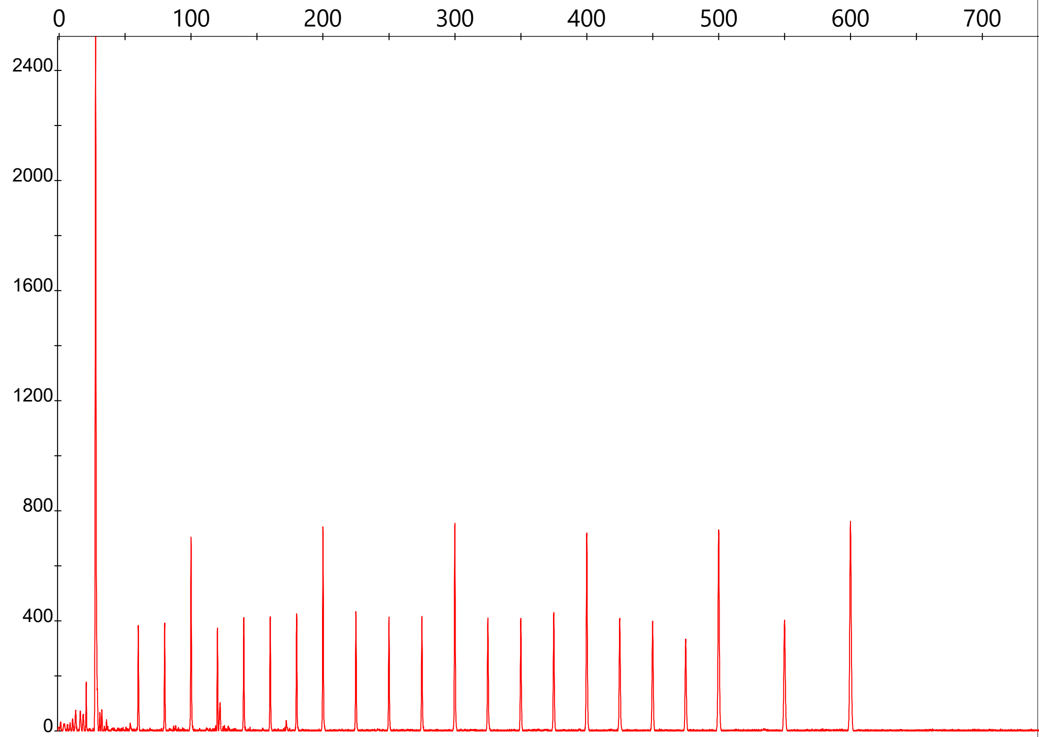

Legend

Dye colours – Correspondig fluorophores (Microsatellite loci)

- ■ Tamra (AK01, AK09, AK13)
- ■ Hex (AK05, AK10, AK15)
- ■ 6-Fam (AK03, AK07, AK12, AK23, AK28)
- ■ Marker ILS600 (Promega)

Multiplex Assays

MultiA: AK01, AK05, AK07, AK13
MultiB: AK03, AK09, AK10, AK12
MultiC: AK15, AK23, AK28
